# Targeted Secure Messages to Facilitate Access to Tobacco Treatment Counseling for Veterans: Feasibility Study

**DOI:** 10.2196/mental.7957

**Published:** 2018-03-05

**Authors:** Shaun Shahani, Pearl Korenblit, Pauline Thomas, Marian R Passannante, Richard Carr, Lynn Davis

**Affiliations:** ^1^ Rutgers New Jersey Medical School Newark, NJ United States; ^2^ Veterans Affairs New Jersey Health Care System East Orange, NJ United States; ^3^ Rutgers School of Public Health Newark, NJ United States

**Keywords:** secure messaging, tobacco use, smoking cessation

## Abstract

**Background:**

Studies show that combining nicotine replacement therapy (NRT) with tobacco treatment counseling is most effective for smoking cessation. However, tobacco treatment counseling has been underutilized across the nation. A secure email message sent to patients already taking NRT was hypothesized to increase the utilization of tobacco treatment counseling among Veterans in New Jersey. Secure messaging for communication between patients and providers was implemented through a web-based password-protected, secure messaging account, where Veterans get notified through their personal email when they have a message awaiting them.

**Objective:**

The main objective of this project was to determine if there was a significant increase in adoption of tobacco treatment counseling among Veterans who received a secure message describing the options for tobacco treatment counseling available to them. Secondary objectives were to demographically characterize Veterans who were and were not enrolled in secure messaging, as well as those who opened or did not open a message. Finally, because the language and content of the messages were changed across project phases, this project also sought to determine (by analysis of response rates) the type of language that was most effective at eliciting a response.

**Methods:**

Over two phases, messages were sent to two samples of Veterans prescribed NRT within the prior 90 days of each phase. In phase 1, one message was sent in December 2015 (message 1). In phase 2, one message was sent in July 2016 (message 2) and the same message (message 3) was resent in August 2016 to persons who did not open message 2. Messages 2 and 3 were more directive than message 1. Response rates to message 1 versus message 2 were compared. A logistic regression analysis determined effect of age and gender on enrollment in secure messaging across both phases. The effectiveness of each phase at increasing tobacco treatment counseling was analyzed using a McNemar test.

**Results:**

Message 2, sent to 423 Veterans, had a significantly higher response rate than message 1, sent to 348 Veterans (18%, 17/93 vs 8%, 6/78, *P*=.04). Phase 2 (ie, messages 2 and 3) significantly increased utilization of tobacco treatment counseling (net increase of six tobacco treatment counseling adopters, *P*=.04), whereas phase 1 (ie, message 1) did not (net increase of two tobacco treatment counseling adopters, *P*=.48). Women (odds ratio [OR] 1.6, 95% CI 1.1-2.3) and those aged 30 to 49 years (compared to other age groups) were more likely to be enrolled in secure messaging. Gender and age were not significant predictors of opening or replying to either message.

**Conclusions:**

Although the effect was small, secure messaging was a useful modality to increase tobacco treatment counseling. Directive content with a follow-up message appeared useful. Female Veterans and/or Veterans aged between 30 and 49 years are more likely to use secure messaging.

## Introduction

The combination of medication, typically nicotine replacement therapy (NRT), and counseling (whether in person or by telephone) has been shown to be the optimal method for smoking cessation treatment [1-3]. In a counseling session, the smoker’s motivation for cessation can be strengthened and he/she can learn about the mechanism and proper usage of the medicine [1]. However, in spite of the benefit of counseling in addition to medication, a majority of smokers attempt to quit without using counseling [1]. This lack of utilization of counseling was also apparent at the VA New Jersey Health Care System (VANJHCS), forming the basis for this project.

Generally, smoking rates are higher in Veterans than non-Veterans (29% vs 24%) in people aged 25 to 64 years [4]. One reason for the underutilization of tobacco treatment counseling at VANJHCS may be Veterans’ lack of awareness of the counseling options being offered. At VANJHCS, these options, both of which are facilitated by a certified tobacco counselor, involve either attending weekly group classes or having a one-on-one session (in person or by telephone). In the past, efforts by VANJHCS to reach out to Veterans to inform them about available tobacco treatment counseling were mainly carried out by telephone, requiring excessive effort and time to call Veterans individually, often finding they were not available to be reached by telephone at the time of the call. The implementation of a mass secure email to Veterans already prescribed NRT or Chantix (a medication that helps people to quit smoking) was considered in this project as a more feasible and efficient way to increase awareness and utilization of tobacco treatment counseling in the Veteran population.

The VANJHCS, serving approximately 50,000 New Jersey Veterans, utilizes a system of secure messaging to facilitate communication between patients and providers. Approximately 14,000 Veterans, or 28% of the total Veterans in VANJHCS, are enrolled in secure messaging. Veterans are notified through their personal email when they have a message awaiting them on their password-protected, secure messaging account. One benefit of such a system is that one can, with a single mass email, reach a relatively large number of Veterans and identify how many actually opened the message (ie, clicked on the title of the message to read it).

Use of email communication to promote healthy behaviors has been studied at length [5,6], but not specifically to increase utilization of tobacco treatment counseling. Advantages of email usage in health care include convenience, rapidity of communication, enhanced access, and likely cost-effectiveness [7]. This project evaluated whether messages sent using the secure messaging modality would help to facilitate Veterans’ access to behavioral tobacco treatment therapy sessions that are available at VANJHCS. The group of Veterans who were solicited were those already on NRT and other tobacco treatment medications, such as Chantix. The messages provided the Veterans with information on the times of existing tobacco treatment classes being offered at the VA and also gave the Veterans an opportunity to request a telephone call by a tobacco treatment counselor or to call the tobacco treatment counselor directly. In short, the messages were designed to give the Veterans information on how to access the necessary behavioral therapy component of tobacco treatment. Whether or not they were successful in doing so was determined by measuring the proportion of Veterans who utilized the VA-offered counseling after having received the message. Furthermore, because the language and number of messages sent changed across project phases, this project also sought to determine the optimal strategy in regards to increasing counseling adoption rates.

Another analysis performed in this project involved demographically characterizing Veterans who were and were not enrolled in secure messaging, as well as those who opened or did not open a message. Knowing the population being reached by secure messaging can be a vital piece of information because these findings can clue in administrators and providers on whom to target messages to and whom to target for interventions aimed at increasing secure message familiarity and usage.

## Methods

### Inclusion/Exclusion Criteria

All Veterans in VANJHCS who were currently taking NRT or Chantix were included in the study. “Currently” was defined as within 90 days before each phase because this time frame was expected to identify people who recently initiated or refilled their NRT or Chantix. Veterans taking the drug bupropion were excluded from this study because there are multiple indications for this drug, only one being tobacco treatment.

For the response rate analysis as well as the analysis of the effect of messages on counseling utilization, only those who had been prescribed NRT or Chantix and who were enrolled in secure messaging could be included in the analysis because only those enrolled in secure messaging were sent messages.

### Description of Phases

**Figure 1:**
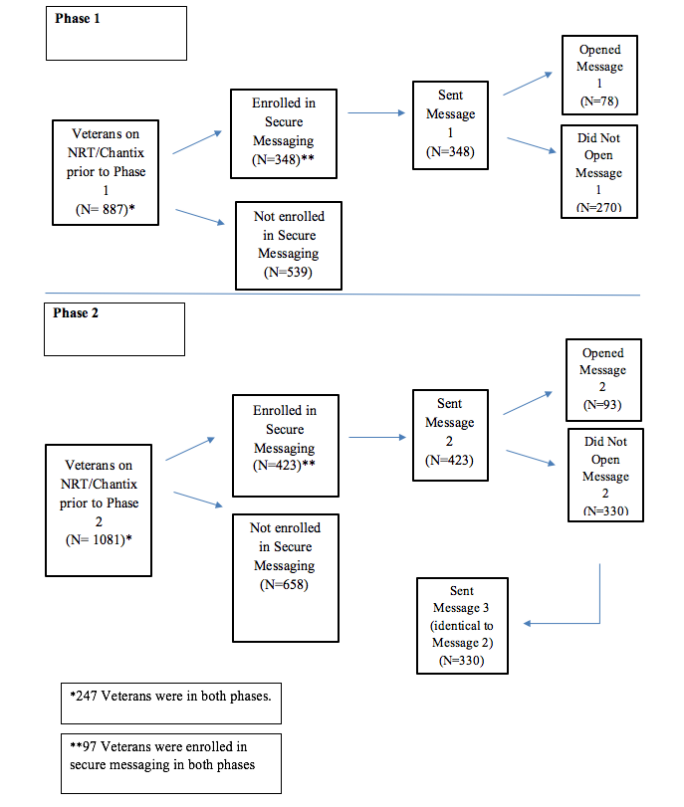
Flowchart illustrating both phases of project.

A total of three secure messages were sent out in two phases. Phase 1 consisted of one message being sent in December 2015 (message 1). Phase 2 consisted of two identical messages being sent in July (message 2) and August (message 3) 2016. Message 3 was sent to every Veteran who was sent but did not open message 2. See Figure 1 for a flowchart illustrating the two project phases. Veterans who were on NRT/Chantix prior to both phases and enrolled in secure messaging in each phase were sent both message 1 and message 2 (with or without message 3 depending on if they opened message 2). The contents of both messages 1 and 2 are provided in Multimedia Appendix 1. The messages were an invitation to attend one of many group tobacco treatment classes offered in the VANJHCS. They also gave the Veteran an opportunity to express interest in receiving individualized phone or in-person counseling by a tobacco counselor.

Message 1 focused on providing information. Veterans were informed about the available tobacco counseling classes, and motivating and supportive language was used. Veterans were not specifically told to respond back with a specific counseling option preference, but they were advised to feel free to contact us so that we could assist them.

Message 2 was more directive. Veterans were given a multiple choice of counseling options and asked (both in the subject line and in the text) to select and then let us know which option they would like to pursue.

### Data Collection

The list of Veterans who were currently on NRT or Chantix (ie, within 90 days prior to each phase) was provided by the pharmacy department at VANJHCS. As explained previously, there were two phases of sending out messages, one in December 2015 and the other in July and August 2016. Before each of the two phases, a current list (ie, Veterans prescribed NRT or Chantix within the prior 90 days) was obtained. For the demographic analysis, the electronic medical record of each unique Veteran on NRT or Chantix was reviewed to abstract age and gender of the Veteran. Whether each Veteran on NRT or Chantix was enrolled in secure messaging and whether a message was opened or responded to (within 30 days after the message was sent) was identified on the VA internal secure messaging website. “Responding” to a message was defined as replying back to our message with another message within 30 days after our message was sent. Whether or not a Veteran was in counseling within the prior 90 days of each phase was determined from the electronic medical record at the outset of each phase by noting if there was a “smoking cessation note” in their electronic medical record. Whether counseling was adopted after each message was sent was reviewed 1 month following the message by a similar method. All these data were input into an Excel spreadsheet and then imported into SAS for analysis.

### Data Analysis

Analysis was done using SAS at an alpha level of .05. The demographic analysis consisted of both bivariate and multivariate analyses. For the bivariate analysis, two-sample *t* tests determined (1) the difference in mean age of Veteran secure messaging enrollees versus nonenrollees and (2) the difference in mean age of enrollees who opened versus did not open each message. Chi-square (in the case of males) and Fisher exact tests (in the case of females due to small sample size) examined whether gender was associated with enrollment in secure messaging or opening a secure message.

For the multivariate analysis, a logistic regression analysis was performed with age and gender as the two covariates studied, and with three binary (yes/no) outcome variables: being enrolled in secure messaging, opening the message (among enrollees), or responding to the message (among message openers). For the latter two outcomes, because the language of messages 1 and 2 were different, the logistic regression was run separately for each message. When considering the outcomes of opening or responding to message 2, Veterans who had also been sent message 1 were excluded to account for any potential effect that receiving message 1 might have had on opening or responding to message 2. For all these outcomes, interaction effects were investigated between gender and age.

In the logistic regression analysis mentioned previously, age was measured first as a continuous variable; however, another logistic regression analysis was run coding age as a categorical variable. This was done because it was noted that secure message enrollment varied markedly by 10-year age group. The 10-year age groups with the highest percent enrollment in secure messaging were ages 30 to 39 (45.6%, 82/180) and 40 to 49 (46.1%, 76/165). Because of the similarity in percent enrollment between these two groups, one 20-year age group, namely 30 to 49 years, was created and used as the reference group to which other 10-year age groups were compared.

To analyze the message response, a two-proportion *z* test was performed to see if the response rate (among message openers) was significantly different between message 1 and message 2. To measure message effect on utilization of tobacco cessation counseling, 1 month after each phase (ie, analyzing message 1 versus both messages 2 and 3 in tandem), a McNemar test analyzed if there was a significant net increase in Veterans entering therapy as a result of opening the message(s). If a Veteran was already in counseling within the prior 90 days of the message, he or she was not counted as a “new” adopter of counseling, whether or not counseling continued after the message was received.

## Results

### Part 1: Demographic Analysis

#### Enrollment in Secure Messaging

There were 1721 unique Veterans who had been prescribed NRT: 640 in December only, 834 in July only, and 247 in both periods. Four were not enrolled in secure messaging prior to message 1 but enrolled prior to message 2 and were thus not included in the demographic analysis. The remaining 1717 had the same secure messaging enrollment status for both phases. Table 1 shows that approximately 39% (670/1717) of Veterans, who had been prescribed NRT or Chantix, were enrolled in secure messaging.

Of the 1717 Veterans, 92.02% (n=1580) were male. Age was available for 1586 Veterans and ranged from 22 to 92 years. The mean age was 56.4 (SD 13.5) years, and the median was 59 years. Of the 1586 Veterans, 63.68% (n=1010) were in the age group 50 to 69 years, (39.22%, 622/1586 aged 60-69 years and 24.46%, 388/1586 aged 50-59 years). Of the remaining 36.32% (576/1586), 21.75% (345/1586) were 30 to 49 (11.35%, 180/1586 aged 30-39 years and 10.40%, 165/1586 aged 40-49 years), and 14.56% (231/1586) were divided between 80 years and older (1.83%, 29/1586), 70 to 79 years (8.26%, 131/1586), and 20 to 29 years (4.48%, 71/1586). The mean age for women was 48.4 (SD 11.3) years compared with 57.0 (SD 13.5) years for men.

Mean age of secure message enrollees was 2 years younger than nonenrollees (mean 55.3, SD 13.0 years vs mean 57.0, SD 13.8 years, *P*=.01). In all, 53.2% (75/141) of women were enrolled in secure messaging versus 37.75% (595/1576) of men (*P*<.001).

#### Opening and Responding to Messages

As shown in Table 2, among those enrolled in secure messaging with available age data, the mean ages of those who opened and did not open message 1 was not significantly different (mean 54.0, SD 13.8 years for openers, mean 54.9, SD 12.9 years for nonopeners, *P*=.62). Similarly, the mean ages of those who opened and did not open message 2 was not significantly different (mean 56.3, SD 11.9 years for openers, mean 55.9, SD 12.7 years for nonopeners, *P=*.80). This nonsignificance was retained after excluding the 85 Veterans (with available age data) who were sent message 1.

**Table 1:** Secure messaging enrollment status of Veterans (total).

Enrollment status	n (%)
Enrolled in secure messaging	670 (39.02)
Not enrolled in secure messaging	1047 (60.98)
Total	1717

**Table 2:** Age of Veterans who opened and did not open messages 1 and 2.

Message	Opened the message		Did not open the message		*P* value
	n (%)	Age (years), mean (SD)	n (%)	Age (years), mean (SD)	
Message 1 (n=313)	68	54.0 (13.8)	245	54.9 (12.9)	.62
Message 2 total (n=375)	85	56.3 (11.9)	290	55.9 (12.7)	.80
Message 2 excluding 85 Veterans sent message 1 (n=290)	71	55.3 (12.1)	219	56.0 (13.0)	.69

**Table 3:** Gender of Veterans who opened and did not open messages 1 and 2.

Message and gender		Opened the message, n (%)	Did not open the message, n (%)	*P* value
**Message 1 (n=348)**				.41
	Male	67 (21.8)	241 (78.2)	
	Female	11 (28)	29 (72)	
**Message 2 total (n=423)**				.85
	Male	84 (22.1)	296 (77.9)	
	Female	9 (21)	34 (79)	
**Message 2 excluding 97 Veterans sent message 1 (n=326)**				.73
	Male	68 (23.1)	223 (76.9)	
	Female	9 (26)	26 (74)	

In all, 78 of the 348 Veterans sent message 1 opened it (22.4%). As shown in Table 3, men and women were equally likely to open message 1 (21.8%, 67/308 vs 28%, 11/40, *P*=.41). For message 2, 93 of the 423 Veterans who were sent the message opened it (22.0%). Men and women were equally likely to open this message (22.1%, 84/380 vs 21%, 9/43, *P*=.85). This nonsignificance was retained after excluding the 97 Veterans who were sent message 1.

The proportion of Veterans who responded to message 1 among those who opened message 1 (6/78, 8%) was significantly different from the proportion who responded to message 2 among those who opened message 2 (17/93, 18%, *P*=.04). The proportion of Veterans who opened message 1 (22.4%, 78/348) was not significantly different from the proportion that opened message 2 (22.0%, 93/423, *P*=.89).

Of the 19 responses to messages 2 and 3, when Veterans did choose an option for counseling, they favored receiving a phone call (5/19) or attending group in addition to receiving a phone call (1/19) more so than attending group alone (0/19). Of the remaining 13 Veterans not choosing a counseling option, five indicated that they had already quit, six indicated they would like to quit on their own without the aid of counseling, one Veteran indicated that he is already in group counseling, and one Veteran responded by asking if he could use his nicotine lozenges with his dentures. One of the Veterans who said he would like to quit on his own also used the opportunity of being sent a message to request a refill for his nicotine gum.

#### Logistic Regression Analyses

As shown in Table 4, when coding age as a continuous variable, female gender (OR 1.61, 95% CI 1.10-2.35), but not age (OR 0.99, 95% CI 0.99-1.00), was a significant predictor of being enrolled in secure messaging. However, once a Veteran was enrolled in secure messaging, neither gender nor age were significant predictors of opening either message 1 or message 2 (analysis conducted separately for both messages). Also, once a Veteran opened a message, whether he/she responded was independent of age and gender. There were also no significant interaction effects between age and gender for all outcomes mentioned previously (not shown).

**Table 4:** Logistic regression: odds ratios (with 95% confidence intervals) for the effect of age (as continuous variable) and female gender on different outcomes.

Variable	Outcomes, OR (95% CI)						
	Being enrolled in secure messaging (yes/no)	Opening message 1 (yes/no)	Opening message 2 (yes/no)	Opening any message^a^ (yes/no)	Responding to message 1 (yes/no)	Responding to message 2 (yes/no)	Responding to any message^b^ (yes/no)
Female gender	1.61 (1.10-2.35)	1.12 (0.47-2.68)	1.06 (0.43-2.65)	1.09 (0.58-2.04)	78.31 (0.75-999.99)	1.14 (0.11-11.34)	2.49 (0.41-15.08)
Age	0.99 (0.99-1.00)	0.99 (0.98-1.02)	1.00 (0.98-1.02)	1.00 (0.98-1.01)	1.18 (1.00-1.39)	1.03 (0.97-1.09)	1.05 (0.99-1.11)

^a^Among Veterans who received only message 1 or only message 2 (n=603).

^b^Among Veterans who opened only message 1 or only message 2 (n=139).

**Table 5:** Logistic regression: odds ratios (with 95% confidence intervals) for the effect of age category and female gender on enrollment in secure messaging.

Covariates		Enrolled in secure messaging (yes/no), OR (95% CI)
Female gender		1.57 (1.07-2.30)
**Age group (years)^a^**		
	20-29	0.41 (0.23-0.73)
	50-59	0.73 (0.54-0.98)
	60-69	0.74 (0.57-0.97)
	70-79	0.62 (0.40-0.94)
	≥80	0.09 (0.02-0.40)

^a^Reference category: 30-49 years.

The preceding results pertain to age measured as a continuous variable. However, after coding age as a categorical variable with the age group 30 to 49 years as the reference category (Table 5), significance was also found for the effect of female gender on secure messaging enrollment (OR 1.57, 95% CI 1.07-2.30). Additionally, the following age groups were significantly less likely to be enrolled in secure messaging compared to those aged 30 to 49 years: 20 to 29 years (OR 0.41, 95% CI 0.23-0.73), 50 to 59 years (OR 0.73, 95% CI 0.54-0.98), 60 to 69 years (OR 0.74, 95% CI 0.57-0.97), 70 to 79 years (OR 0.62;, 95% CI 0.40-0.94), and 80 years or older (OR 0.09, 95% CI 0.02-0.40). After coding age as a categorical variable, odds ratios for opening and responding to either message did not change dramatically from before and are thus not included in this report.

### Part 2: Analysis of Effect of Message on Increasing Counseling Utilization

McNemar tests were run after each phase of messaging to observe if the net increase in counseling adopters significantly increased. For message 1, although 78 Veterans opened the message, 10 charts were not accessible and were thus conservatively assumed to not have adopted counseling. Among the 68 (with accessible charts) who opened the message, only two additional Veterans adopted counseling (net increase from 4 to 6, *P=*.48).

Phase 2 (423 total Veterans sent message 2 or both messages 2 and 3) consisted of message 2 (93 message openers) and message 3 (21 additional message openers), for a total of 114 message openers for the entire phase. Of these, eight had charts that were not accessible and were thus conservatively assumed to not have adopted counseling. Among 106 Veterans with readily accessible charts, six new Veterans adopted counseling and this was statistically significant (net increase from 11 to 17, *P=*.04). Five of these six counseling adopters did so after having opened message 2, whereas one of the six adopted counseling after opening message 3. None of these six counseling adopters received message 1.

## Discussion

This study examined use of secure messaging in VANJHCS to increase utilization of tobacco treatment counseling. A directive message (plus its identical follow-up message) resulted in a small but significantly greater number of new counseling enrollees. Female gender was found to be a significant predictor of enrollment in secure messaging even when controlling for age. Although not entirely comparable, this finding may be related to conclusions from previous literature that have found that women utilize health care more than men [8,9]. Messages directed toward females (eg, breast cancer screening reminders or osteoporosis screening reminders) might be more amenable to secure messaging.

Although age when measured as a continuous variable was not a significant predictor of enrollment in secure messaging, after combining age groups with similar enrollment percentages and adjusting for gender, those in the age group with the highest percentage of enrollment (30-49 years) were more likely to be enrolled in secure messaging than other age groups. Also, except for the 20 to 29 age group, there does seem to be an association between increasing age (especially after age 50) and lack of enrollment in secure messaging. Secure messaging might not be as viable an option for those older than age 50. Future studies should attempt to identify specific barriers to using secure messaging in the older population. The association between increasing age and lack of enrollment was likely not noticed at first (when age was measured as a continuous variable) because of the 20 to 29 years age group having a disproportionate number of nonenrollees despite their young age. Determining reasons for lack of enrollment in this age group is a subject for further research. A notable limitation regarding the conclusions reached regarding demographic characteristics associated with secure messaging enrollment is that our sample only consisted of Veterans on NRT, which may or may not be representative of the population of Veterans who do not use NRT. Moreover, because of lack of access to data such as education level and socioeconomic status, we were unable to analyze the effect of these other factors on secure messaging enrollment.

The increase in tobacco counseling encounters was not significant after the first message was sent out (phase 1), but it was significant after the second and third messages (phase 2). Additionally, because none of the counseling adopters after the second and third messages had received message 1, this was a true measure of the effect of these messages. However, although being statistically significant, the net increase in counseling utilization was very small (only six Veterans). This is likely because of the low overall enrollment in secure messaging and the fact that the rate of opening the messages (for both the first and second phases) was low. We must look beyond demographic variables to explain this latter phenomenon because it was determined that age and gender were not statistically significant factors associated with opening a message. Future studies should explore other reasons why Veterans did not open the messages, such as uninteresting subject lines, lack of interest or motivation to click on a message having to do with tobacco (subject lines for both messages included the word “tobacco”), infrequent email checking, receiving too many emails, or lack of technical skill required to open a message in one’s inbox. Compared to message 1, the increase in response rate to message 2 (which was more directive in nature and contained a multiple-choice option that Veterans were requested to choose from) may have played a part in the significant increase in tobacco counseling utilization after phase 2. Another implication is that the sending of an identical follow-up message to those who did not open a preliminary message, as was done in phase 2, may be an effective approach. A limitation of our study was the seasonal difference in the timing of the phases (winter for phase 1, summer for phase 2), which may have led to the increased response rate to message 2 compared with message 1. Future studies should attempt to control for seasonal variation by comparing response rates to messages that were sent in the same season (of consecutive years).

In trying to determine which phase was associated with increased tobacco counseling enrollment, there were notable limitations in both the choice of our outcome measure and predictor variables. Regarding outcomes, an obvious limitation lies in what we used as our outcome measure, namely enrollment in tobacco cessation counseling, with “success” being defined as a net increase in enrollment. It would have been informative if we had an outcome measure on actual quit rates, which although not used in this study due to a lack of access to that data, would be advantageous to include in future studies. As far as addressing what drove Veterans to adopt tobacco cessation counseling, in our study we focused on the attributes of the messages themselves (ie, comparing differing content and number of messages sent in phase 1 vs phase 2). In future studies, among those who do adopt counseling, it would be helpful to also look at individualistic factors that may predispose one to adopt counseling, such as baseline intention of quitting and prior history of quitting.

When given several options for counseling (as was the case in messages 2 and 3), Veterans preferred telephone counseling (either alone or in addition to group), although the number of responses was too small to establish significance. No Veteran opted for attending group alone. Knowing that Veterans might be more inclined to utilize telephone counseling can clue in providers to offer this as an option to Veterans. An unexpected benefit from secure messaging was the facilitation of communication between patient and provider. Two Veterans utilized secure messaging as an opportunity to serve other related health interests, such as clarifying if dentures could be worn while using lozenges in the case of one Veteran or to ask for a refill on his nicotine gum in another. Although these Veterans may have had another opportunity to address these issues in the future, secure messaging provided them with a quick and convenient way to communicate these concerns with someone who was willing to listen.

The increases in tobacco counseling utilization in this study were achieved by using an economical and efficient approach (ie, secure messaging). An argument can be made that the cost-effectiveness and convenience of secure messaging make it a favorable action when compared with other alternatives of outreach, such as telephone calling each smoker who is on NRT or Chantix, which may not be practical given limited time and resources. However, the total number adopting counseling was quite small in this initial feasibility study and further research is needed to better understand both how to increase rates of enrollment in secure messaging and also to improve the effectiveness of messages.

## Appendix

Multimedia Appendix 1 Content of the messages.

## Abbreviations

NRT: nicotine replacement therapy
VANJHCS: Veterans Affairs New Jersey Health Care System
