# Supplementary material for: Targeted Secure Messages to Facilitate Access to Tobacco Treatment Counseling for Veterans: Feasibility Study
Source: JMIR Ment Health. 2018 Mar 5;5(1):e18. doi: 10.2196/mental.7957 (PMC5859788; doi:10.2196/mental.7957)
Supplement: Multimedia Appendix 1 [file mental_v5i1e18_app1.pdf]

Content of the Messages

Message sent in December 2015 (Message 1)

Subject line: Tobacco Medication Follow-up
Dear Veteran,
You are receiving this message because you recently expressed an interest in cutting down or quitting smoking.

We commend your choice to take medications to help reduce or eliminate tobacco use. Other veterans that we worked with were able to quit with help from us and using tobacco medication.

You can QUIT SMOKING AT YOUR OWN PACE with help from the VA.

Please let us know if you would like to be called for help in quitting smoking, or let us know if you quit already!!!!!

We also have groups you can attend!!
See attachment for a list of all available timings and locations of the tobacco groups at the VA.

Feel free to contact xxxxx at (973) xxx-xxxx, and leave a message so that she can call you back and she can assist you.

Quitting is hard but not impossible.

Sincerely,
HPDP Staff

P.S. You are welcome to refer to the attached handout describing "Benefits of Quitting."

Messages sent in July and August 2016 (Messages 2 and 3)

Subject line: Tell us which tobacco counseling option you would like!
Dear Veteran,

You are receiving this message because your records indicate that you are taking medication (such as the nicotine patch, gum, lozenge, etc.) to help you quit smoking.

In addition to taking medication, it is the combination of medication PLUS counseling that is most effective to help you quit. The counseling can come in the form of a telephone call. Or, if you prefer, you can attend one of our tobacco groups. The list of all groups offered at the VA is attached to this message.

At your convenience, please reply to this message indicating which tobacco treatment counseling option you'd like from the choices below:

a.) Group -- and if so, which group you want to attend (see attachment)
b.) Phone call -- and if so, what time is best for us to call you
c.) Neither -- "I'll quit on my own."
d.) Not applicable -- "I have quit already" or "I am already in group"
e.) Other

We look forward to hearing back from you. We wish you the best of luck in your attempt to quit smoking.

Have a wonderful day!

Sincerely,
HPDP Staff
